# How Does Job Insecurity Affect Workplace Harassment? The Interaction Effect of Hypercompetitive Attitude, Coworker Impression Management, and Leader Narcissism

**DOI:** 10.3389/fpsyg.2021.753061

**Published:** 2021-10-13

**Authors:** Geunhye Song

**Affiliations:** Technology Policy Research Division, Electronics and Telecommunications Research Institute, Daejeon, South Korea

**Keywords:** workplace harassment, impression management, leader narcissism, hypercompetitive attitude, job insecurity, artificial intelligence

## Abstract

With concerns that artificial intelligence may replace existing jobs, job insecurity is becoming more prevalent. In-depth study of how job insecurity affects our society has become an important research topic. This study investigates the internal mechanisms through which such job insecurity influences workplace harassment. Based on the theories of psychological contract breach and the conservation of resources, this study proposes an indirect effect of job insecurity and a three-way moderation effect of hypercompetitive attitude, perceived coworker impression management, and leader narcissism on aggression intention. Using survey data from 286 employees in South Korea, bootstrapping analysis, hierarchical regression analysis, and a slope-difference test were performed to confirm the mediation and moderation effects. The results showed that hypercompetitive attitude mediates the association between job insecurity and aggression intention. The three-way interaction effect was also confirmed, such that the interaction effect of hypercompetitive attitude and coworker impression management is only effective when leader narcissism is high. This study contributes to the literature and business practices by offering significant suggestions to aid a more in-depth understanding of the workplace harassment occurrence process.

## Introduction

Many jobs can now be substituted by artificial intelligence (AI), causing job insecurity to become more widespread: Globally, about 60% of employees are at risk of unemployment due to the increasing proliferation of AI (McKinsey and Company, 2019), which is expected to replace both standardized and unstructured jobs (Frey and Osborne, 2017). This implies that not only simple and repetitive tasks but also professional functions are likely to be replaced, in a situation that reminds us of the industrial revolution, where new technologies such as the steam engine, electricity, and ICT technology drove changes in the employment environment (Schwab, 2016). Before new jobs were created and generalized by these technologies, many employees were unemployed and underwent vocational retraining to acquire new skills. During this process, various forms of social conflicts arose, and the advancement of AI is believed to bring about a similar pattern (European Parliamentary Research Service, 2019): In the long term, new jobs will be created by AI, but in the short term, it is anticipated that diverse issues will likely occur. Job insecurity is an unavoidable phenomenon in the current business environment, and almost all employees will be forced to experience it (Lee et al., 2018).

Job insecurity is an individual employee's subjective perception, defined as “perceived powerlessness to maintain desired continuity in a threatened job situation” (Greenhalgh and Rosenblatt, 1984). It occurs in the current job environment and differs from real unemployment since it is characterized by uncertainty about the future (Huang et al., 2013). Moreover, even in the same situation, the level of job insecurity experienced by each individual employee can vary widely (Greenhalgh and Rosenblatt, 1984; Ashford et al., 1989). Previous studies have found evidence supporting the negative effects of job insecurity; for example, job insecurity has been shown to negatively affect organizational commitment, job satisfaction, job performance, and employee health (Ito and Brotheridge, 2007; Wang et al., 2015; Jiang and Probst, 2019). Empirical research on job insecurity has increased over the last 15 years; however, few studies have yet analyzed the impact of job insecurity on deviant behavior in the workplace (Schilpzand et al., 2016; Lee et al., 2018). Thus, the literature has called for further investigation into the psychological processes that influence the relationship between job insecurity and workplace harassment (Huang et al., 2017; Lee et al., 2018).

Workplace harassment is “a general term encompassing all forms of behavior by which individuals attempt to harm others at work of their organizations” (Neuman and Baron, 1998). In the literature, workplace harassment has been conceptualized via a variety of labels, including “incivility,” “interpersonal conflicts,” and “workplace bullying,” which are very similar constructs (Hershcovis, 2011). Even among various variables of workplace harassment, “intent to harm” is a common prominent key feature, which distinguishes deliberate aggression from accidental and unintended actions (Goldsmid and Howie, 2014). In line with this concept, the present study investigates workplace harassment by concentrating on aggression intention.

Previous studies have examined the antecedents of workplace harassment from various levels; for example, research about individual attributes such as the “Big Five”; stressful job characteristics including workload, role ambiguity, and job insecurity; organizational characteristics such as lack of justice have been undertaken (Hackney and Perrewé, 2018; Nielsen and Einarsen, 2018; Rai and Agarwal, 2018). Although limited research has examined the relationship between job insecurity and workplace harassment, job insecurity was shown to be one of the most powerful antecedents of workplace harassment in one review study (Van den Brande et al., 2016). However, previous studies have mostly focused on the direct effect of job insecurity on workplace harassment, and few have investigated the underlying mechanism and the aggravating or alleviating factors in the relationship between the two variables. Additionally, the literature has recently called for a study that discloses the in-depth mechanism of workplace harassment from a perpetrator's perspective (Nielsen and Einarsen, 2018; Rai and Agarwal, 2018). Recognizing this call and fulfilling the research gap, this study aims to analyze the association between job insecurity and workplace harassment more thoroughly using mediating and moderating variables and employing “psychological contract breach theory” and “conservation of resource (COR) theory” as a theoretical background.

In particular, this study suggests that aggression intention is encouraged by job insecurity because employees feel a psychological contract breach with their organization and, subsequently, struggle to conserve their resources to sustain their position; that is, the hypercompetitive attitude induced by job insecurity can prompt the possibility of aggression intention. Moreover, this study assumes that two contextual factors can reinforce the effect of hypercompetitive attitude on aggression intention: perceived coworker impression management (IM) and leader narcissism. Specifically, the more coworkers are perceived to be threatening to their peers but sympathetic to their leader, the stronger the effect of the hypercompetitive attitude on the aggression intention. Leader narcissism may act as a signal to allow such situations, enabling employees to be more blatantly competitive and making them more likely to involve in aggression intention.

Previous studies have not provided sufficient evidence as to how the effects of job insecurity occur (Lee et al., 2018), and almost all previous studies that have applied COR have been addressed in the stress literature (Hobfoll et al., 2018); however, this study uses COR theory to empirically examine the association between job insecurity and aggression intention, thus extending the scope of this theory.

## Theoretical Background

**Figure 1:**
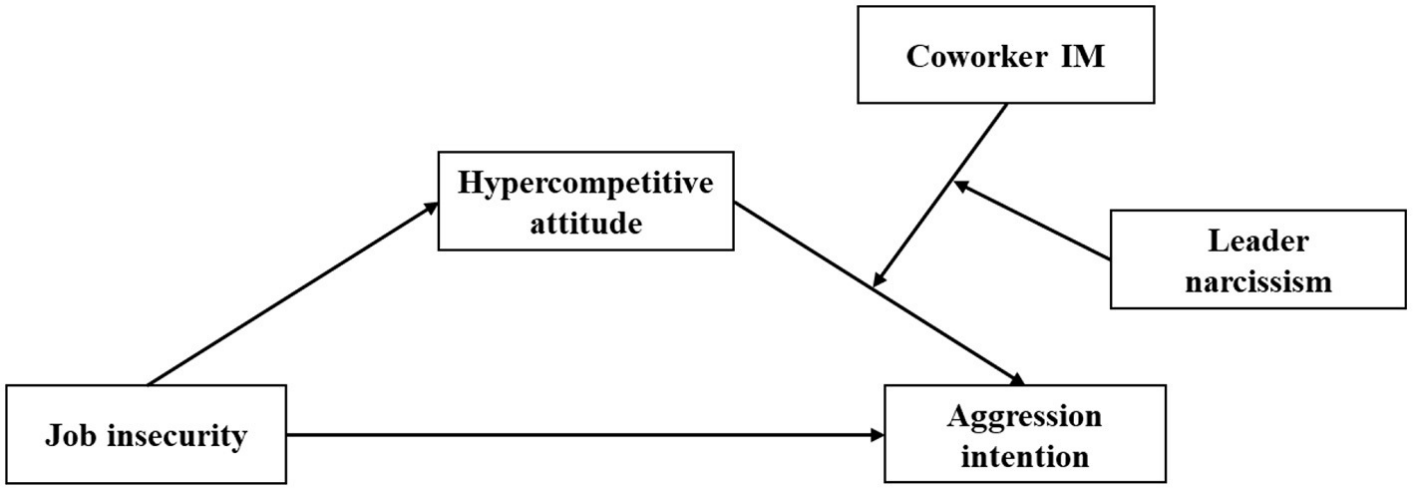
The research model.

This study investigates the relationship between job insecurity and workplace harassment by integrating the theories of psychological contract breach and conservation of resources. First, this study assumes that job insecurity is associated with aggression intention via hypercompetitive attitude. Second, this study focuses on the three-way interaction effect of hypercompetitive attitude, coworker IM, and leader narcissism on aggression intention. The proposed model is shown in Figure 1.

### Job Insecurity and Aggression Intention

Psychological contracts, i.e., implicit, unwritten, and informal mutual obligations, are formed between organizations and their employees (Argyris, 1960). Organizations implicitly expect commitment, dedication, and loyalty from their employees, whereas employees anticipate obligations, recognition, rights, and job security from organizations. However, when a psychological contract is violated, employees experience negative feelings about their organization, such as anger, frustration, and dissatisfaction, believing that the organization has not met its promises. Such feelings can affect employees' attitudes and behaviors, which, in turn, can have a detrimental effect on both an employee themselves and the organization.

Job insecurity can be seen as a violation of psychological contracts (De Cuyper and De Witte, 2007): In general, employees expect their organization to ensure job security. However, when employees feel they are at risk of becoming unemployed, they believe that the organization has breached these tacit agreements. If employees feel that a psychological contract has been violated, they are more likely to exhibit harmful behaviors, such as turnover intention, absenteeism, workplace bullying, and deviant attitudes (Pate et al., 2003; Suazo, 2009; Rajalakshmi and Naresh, 2018). In particular, employees who experience job insecurity are likely to lower their dedication to the organization and relinquish their associations with it (Ashford et al., 1989; Bernhard-Oettel et al., 2011; Lee et al., 2018).

Based on this psychological contract violation theory, job insecurity can affect the formation of negative employee attitudes and behaviors. This study analyzes the formation process of the aggressive intentions of workers who experience job insecurity, specifically focusing on the mechanism of aggression intention before selecting targets.

### Mediation Effect of Hypercompetitive Attitude

This study suggests that the aggressive response of employees experiencing job insecurity is mediated by hypercompetitive attitude, based on COR theory. According to the theory, employees make endless efforts to seek, secure, and maintain important resources and feel threatened when faced with losing, being at risk of losing, or unlikely to obtain such resources, which leads to employees' desire to remove themselves from the situation.

In COR theory, “resources” refers to the objects, personal characteristics, conditions, and energy that employees value, consisting of lower-ordered resources (e.g., time, energy) and higher-ordered resources (e.g., self-esteem, social status, power). In most cases, employees try to acquire and maintain higher-ordered resources using lower-ordered resources (Hobfoll, 2001). Job security is of value itself as it brings employees higher-ordered resources, such as social status, pride, or self-confidence (Jahoda, 1981); however, those employees who suffer from job insecurity are in a state of threat to potentially or substantially lose these resources. Empirical studies have suggested that employees who experience job insecurity are more likely to undergo burnout, emotional exhaustion, or low self-esteem (Cheng and Chan, 2008).

States of low self-esteem and psychological exhaustion can induce hypercompetitive attitudes since such attitudes reflect a neurotic and exaggerated form of the competitive stance, which is related to low self-esteem (Ryckman et al., 1990). Individuals high in hypercompetitive attitude tend to strongly perceive the need to compete for self-worth; thus, employees are more inclined toward hypercompetitive attitude when experiencing job insecurity, in order to negate lowered self-esteem and retain self-worth. Indeed, employees experiencing job insecurity strive to minimize and restore lost resources, rather than merely observing the situation (Hobfoll, 2001). As such, employees who experience job insecurity are more likely to adopt a hypercompetitive attitude as a way to escape their negative psychological state. Applying COR theory in this situation, “strain” can be expressed as a hypercompetitive attitude.

Individuals with hypercompetitive attitude have a strong need to win to feel superior to others and are acquiescent to using unfair strategies to compete; this tendency is related to the attitude of derogating another party by considering the peer or surrounding situation as harmful to the self (Horney, 1937). Several empirical studies have confirmed that hypercompetitive attitudes positively predict maladaptive outcomes: Specifically, aggression, neuroticism, dogmatism, interpersonal problems, and the negative aspect of perfectionism have all been found to be outcomes of a hypercompetitive attitude (Ryckman et al., 1990, 2002; Orosz et al., 2018). Hence, the effect of job insecurity on hypercompetitive attitude can lead to aggression intention. Based on this logic, this study hypothesizes that employees who feel job insecurity are more likely to have aggression intention via hypercompetitive attitude.

**Hypothesis 1**. Hypercompetitive attitude mediates the positive relationship between job insecurity and aggression intention.

### Interaction Among Coworker IM and Leader Narcissism

Impression management is defined as the behaviors individuals employ to shape their image (Rosenfeld et al., 1995). In organizational settings, employees shape their images through awareness of the way other people view them. Typically, supervisors, coworkers, subordinates, or customers represent those “other people” (Bolino et al., 2016). IM consists of five components: ingratiation, exemplification, intimidation, self-promotion, and supplication (Jones and Pittman, 1982). Generally, ingratiation is categorized as supervisor-focused IM tactics (Bolino et al., 2006), whereas intimidation is related to coworker-focused IM tactics (Lukacik and Bourdage, 2019). In line with this previous research, the present study focuses on IM in terms of perceived intentional behavior by coworkers using ingratiation toward a leader and intimidation toward their peers.

The present study focuses on perceived coworker IM since coworkers are proximal to a focal employee; in most workplaces, people work more directly with their peers, and coworkers can significantly impact the perception, attitude, and behavior of a focal employee. For instance, a focal employee's job satisfaction, commitment, or organizational citizenship behavior has been found to be positively influenced by coworker support, where coworkers' antagonism is negatively related to these variables (Chiaburu and Harrison, 2008).

Coworker IM can be influential to a focal employee. IM entails specific deliberation to influence others, which can be regarded as unethical (Schoderbek and Deshpande, 1996), and is seen as a political behavior by other employees (Bourdage et al., 2015). Such findings indicate that employees perceive their coworkers' IM as an activity intended to create images with a specific purpose. This issue is theoretically significant since the degree to which coworkers are perceived as using IM tactics can strengthen the exclusive behavior of a focal employee who is concentrated on securing self-focused resources.

This study assumes that coworker IM operates as a moderator in the relationship between hypercompetitive attitude and aggression intention. IM by coworkers can negatively influence employees, who expose hypercompetitive attitude. According to previous studies, perception of coworkers' IM decreases a focal employee's well-being and increases dysfunctional ideation and emotional exhaustion (Porath and Erez, 2009; Totterdell et al., 2012). Furthermore, employees who perceive themselves to be unfairly treated by their coworkers are more threatened by sense-making about these stimuli (Green and Mitchell, 1979); therefore, perceived coworker IM can stiffen a focal employee's detrimental intention, especially when exposing a hypercompetitive attitude. Above all, a focal employee might judge the lack of social support in the organization: Recognizing that coworkers use IM can increase a focal employee's aggression intention since IM can politicize the workplace, and IM performed for deceptive purposes can lead to resource misallocation (Ferris and Kacmar, 1992). On the other hand, a focal employee might be less aggressive when their coworkers' IM is not perceived to be prominent. Taken together, although aggression intention arises through hypercompetitive attitude, the effect might be stronger when a focal employee perceives that their coworkers frequently use IM; thus, employees who strongly perceive their coworkers to manage their impressions will be more likely to have aggression intentions.

The workplace harassment literature has emphasized the need to study situational variables within organizations (Hershcovis et al., 2007). Since leadership affects the attitudes and minds of organizational members, the interaction effects of hypercompetitive attitude and coworker IM can be qualified by leadership; that is, some individuals might expose aggression intention since they perceive it as approval. This study posits leader narcissism as a moderator since previous research has indicated that it can influence employees' behavior, even in a morally violated situation (Germain, 2018).

Leader narcissism is characterized as a grandiose sense of self-importance, feelings of entitlement, and self-affirmation (Rhodewalt and Peterson, 2009). The concept is drawn from clinical and personality psychology; however, organizational psychologists have focused on the implicit characteristics of narcissism, rather than evaluating it in terms of mental illness (Braun, 2017). In the leadership narcissism literature, it is assumed that narcissism is relatively stable and is prominent in leaders (De Vries and Miller, 1985). Those high in leader narcissism typically display low empathy ability, continuously desire to be recognized and superior, and manipulate conversation (Judge et al., 2009). Narcissistic leaders are known to have a negative impact on their subordinates: Evidence has indicated that subordinates are more likely to engage in counterproductive work behavior toward people and organizations when they perceive their leaders to be narcissistic (Mitchell and Ambrose, 2007; Braun et al., 2018).

This study theorizes that leader narcissism is likely to empower the interaction effect between hypercompetitive attitude and coworker IM; that is, employees will be more likely to engage in aggression intention when their leaders are narcissistic. A focal employee under such leadership might perceive that detrimental behavior is endorsed when seeking resources like self-worth because it is displayed by their leaders; thus, employees are likely to think that aggressive behavior is acceptable for resource acquisition. As a result, the interaction effect between hypercompetitive attitude and coworker IM on aggression intention is stronger for those who perceive the presence of high leader narcissism within their organization. In contrast, this study predicts that employees will discern deviant workplace behavior as dissent when their leaders are less narcissistic, which results in a weaker association between hypercompetitive attitude and aggression intention, even when coworkers employ IM.

Consequently, this study proposes that the effect of coworker IM on the relationship between hypercompetitive attitude and aggression intention is dependent on leader narcissism since such leadership acts as an aggravation toward employees' deviant behavior.

**Hypothesis 2**. A three-way interaction among hypercompetitive attitude, coworker IM, and leader narcissism predicts aggression intention, such that the amplification effect of coworker IM on the relationship between hypercompetitive attitude and aggression intention is stronger for those perceiving higher (as opposed to lower) leader narcissism.

## Method

### Data Collection and Respondents' Characteristics

Data were collected from Korean workers by administering a survey via a specialized research company named World Survey. This research company has a large number of panels recruited through the stratified sampling method based on the Korean national census, and has customers in Korean government offices, universities, and corporations. Surveys were distributed to individual employees by email or smartphone application, and they voluntarily decided to complete the questionnaire. A cover letter accompanied the survey, which stressed the confidentiality and anonymity of responses and gave assurance that there were no right or wrong answers. Independent and dependent variables as well as similar questionnaire items were presented separately to mitigate common method bias (Podsakoff et al., 2012). Participants were rewarded with a coffee coupon upon survey completion. Following the suggestion of Tabachnick et al. (2007), the survey was closed when 300 samples were obtained. As a result, valid data from 286 participants were extracted after excluding missing data and outliers. Table 1 describes the respondents' characteristics.

**Table 1:** Survey respondents' demographics.

**Variable**	**Category**	***N***	**Percentage (%)**
Gender	Male	177	61.9
	Female	109	38.1
Age	20s	53	18.5
	30s	116	40.6
	40s	70	24.5
	≥50	47	16.4
Education	High school graduate	26	9.1
	Vocational school	33	11.5
	Bachelor's degree	200	69.9
	Above Master's degree	27	9.4
Employment type	Permanent	263	92.0
	Temporary	23	8.0
Industry type	Production/Technical work	19	6.6
	Office work	214	74.8
	Service/Sales	29	10.1
	R&D/Science	22	7.7
	Other	2	0.7
Tenure	≤1 year	34	11.9
	1–3 year	60	21.0
	3–10 years	120	42.0
	≥10 years	72	25.2

*N = 286*.

### Measures

Since the original questionnaires were written in English, each item was translated into Korean and checked by a bilingual person, and then retranslated by another bilingual person. The Korean version of scale was completed via the translation and retranslation process.

### Job Insecurity

Following prior research (e.g., Hewlin et al., 2016), this study used a self-report scale developed by Ashford et al. (1989) to measure job insecurity (α = 0.76). Respondents were asked to rate to what extent they perceived the threat of a total job loss that might occur in their current position. For example, one sample item was, “I may lose my job and be moved to a lower level within the organization,” which respondents were asked to rate on a 5-point scale ranging from 1 (very unlikely) to 5 (very likely).

### Hypercompetitive Attitude

Hypercompetitive attitude was assessed using Ryckman et al.'s 1990 scale (α = 0.70). Respondents were asked to rate how much they agreed with each item, for example, “I find myself being competitive, even in situations which do not call for competition,” which they rated on a 5-point scale ranging from 1 (never true of me) to 5 (always true of me).

### Aggression Intention

The 12 items developed by Bryant and Smith (2001), which are a short version of Buss and Perry's 1992 scale, were used to measure aggression intention (α = 0.84). Sample items included, “I can't help getting into arguments when people disagree with me,” and “If somebody hits me, I hit back,” which were rated on a 5-point scale ranging from 1 (extremely uncharacteristic of me) to 5 (extremely characteristic of me).

### Coworker IM

This study measured coworker IM in terms of respondents' perceptions of their coworkers. Bolino and Turnley's 1999 IM scale was used and modified to assess intimidation toward peers and ingratiation toward leaders (α = 0.77). Respondents were asked to rate how frequently they had perceived that their coworkers used these strategies at work in the last 6 months. Sample items included, “My coworker(s) let colleagues know that they can make things difficult for them if they push them too far,” and “My coworker(s) compliments our manager so the manager will see them as likable,” which were rated on a 5-point scale ranging from 1 (never behave this way) to 5 (often behave this way).

### Leader Narcissism

Narcissistic leadership was assessed using the short version of the Narcissistic Personality Inventory (NPI-13) developed by Gentile et al. (2013) (α = 0.77). This scale asked participants to rate how much they agreed that each statement represented their leader. Sample items included, “My leader likes having authority over other people,” assessed on a 5-point scale ranging from 1 (strongly disagree) to 5 (strongly agree).

### Control Variables

This study controlled for demographic variables such as gender, age, education, employment type, industry type, and tenure since these characteristics could influence the dependent variable (Ng and Feldman, 2015).

## Results

Scale validity was tested using AMOS 21, before the hypotheses were verified. A confirmatory factor analysis (CFA) was performed to examine whether each variable (job insecurity, hypercompetitive attitude, aggression intention, coworker IM, and leader narcissism) was distinctive. The results showed that the five-factor model had a good fit for the data set (*x*^2^ = 102.32 (*df* = 34, *N* = 286), *RMR* = 0.02, *GFI* = 0.94, *CFI* = 0.95, *RMSEA* = 0.08). Next, I checked the average variance extracted (AVE) and composite reliability (CR) to confirm convergent validity. All the standardized λ and AVEs were above 0.68, and CRs were larger than 0.82; thus, the convergent validity of the scales was confirmed. Discriminant validity was also proved, as all AVEs were larger than the squared correlation coefficients of each item, and the correlation confidence interval did not contain one (Fornell and Larcker, 1981; Anderson and Gerbing, 1988). Further, Harmon's one-factor test was performed to avoid common method bias. The result showed that a single factor only explained 26.76% of the total variability; therefore, common method bias was shown to be absent in the data (Podsakoff et al., 2012). Table 2 shows the descriptive statistics and correlations of the variable with internal consistency.

**Table 2:** Descriptive statistics and correlations of variables with internal consistency reliabilities.

**Variable**	**Mean**	**S.D**.	**1**	**2**	**3**	**4**	**5**
1. Job insecurity	3.03	0.65	(0.70)				
2. Hypercompetitive attitude	3.18	0.63	0.47^**^	(0.68)			
3. Aggression intention	3.07	0.63	0.61^**^	0.59^**^	(0.75)		
4. Coworker IM	3.31	0.54	0.53^**^	0.49^**^	0.40^**^	(0.72)	
5. Leader narcissism	3.48	0.56	0.44^**^	0.31^**^	0.40^**^	0.65^**^	(0.78)

*N = 286*.

** *p < 0.01 (two-tailed test)*.

Following these verification results, this study's hypotheses were tested. First, the mediation effect of hypercompetitive attitude was examined using structural equation modeling (SEM). H1 suggested the mediation effect of hypercompetitive attitude between job insecurity and aggression intention. Bootstrapping analysis was used to verify whether the mediation effect was significant, and the results showed that hypercompetitive attitude mediated the relationship between job insecurity and aggression intention (indirect effect = 0.18, 95% CI [0.18, 0.39]). Summarized results of the analysis are shown in Table 3, which indicates the statistical significance of the mediation effect. Hence, hypothesis 1 was supported.

**Table 3:** Bootstrapping results of mediation analysis.

	**B**	**SE**	**t**	**LLCI**	**ULCI**
Total effect	0.60	0.05	13.02	0.5057	0.6859
Direct effect	0.42	0.05	8.88	0.3246	0.5095
Indirect effect	0.18			0.1838	0.3856

*^a^This table is based on 5,000 bootstrap samples*.

*^b^LLCI, lower limit of 95% CI; ULCI, upper limit of 95% CI*.

Next, the three-way interaction effect among hypercompetitive attitude, coworker IM, and leader narcissism was verified using hierarchical regression analysis. H2 postulated that a three-way interaction effect among hypercompetitive attitude, coworker IM, and leader narcissism explains aggression intention. Prior to the analysis, mean centering was conducted for all the predictors to avoid multicollinearity. Variance inflation factor (VIF) was also checked, which showed <2.35; therefore, multicollinearity was not present in the data. Control variables were firstly entered the regression equation. In the second step, hypercompetitive attitude, coworker IM, and leader narcissism were added. In the third step, two-way interaction terms (hypercompetitive attitude and coworker IM, hypercompetitive attitude and leader narcissism, and coworker IM and leader narcissism) were entered. Then, in the final step, a three-way interaction term of hypercompetitive attitude, coworker IM, and leader narcissism was entered. As shown in Table 4, the results revealed that there was a significant three-way interaction effect (*B* = 0.19, *p* < 0.001). Model 4's increment explanatory power significantly increased as per Model 3 (Δ*R*^2^ = 0.02, *p* < 0.05). Since the control variables did not show the statistically significant effects, the impact of the main variables on the dependent variable could be confirmed more clearly.

**Table 4:** Interaction effects of hypercompetitive attitude, coworker IM, and leader narcissism on aggression intention.

**Variables**	**Step 1**			**Step 2**			**Step 3**			**Step 4**		
	**B**	**SE**	**β**	**B**	**SE**	**β**	**B**	**SE**	**β**	**B**	**SE**	**β**
Constant	2.93	0.10		3.01	0.07		2.99	0.07		2.96	0.07	
Employment type	0.06	0.08	0.05	0.07	0.06	0.06	0.05	0.06	0.04	0.04	0.06	0.03
Gender	−0.03	0.04	−0.05	−0.01	0.03	−0.02	−0.00	0.03	−0.00	0.00	0.03	0.00
Education	−0.02	0.04	−0.04	−0.04	0.03	−0.07	−0.03	0.03	−0.05	−0.04	0.03	−0.07
Industry type	−0.07	0.06	−0.08	−0.04	0.04	−0.05	−0.03	0.04	−0.03	−0.03	0.04	−0.04
Age	−0.02	0.03	−0.03	−0.01	0.03	−0.03	−0.01	0.03	−0.02	−0.01	0.03	−0.01
tenure	0.03	0.03	0.06	−0.02	0.02	−0.04	−0.01	0.02	−0.02	−0.00	0.02	−0.01
Hypercompetitive attitude				0.52	0.05	0.52^***^	0.51	0.05	0.51	0.45	0.06	0.44^***^
Coworker IM				0.01	0.08	0.01	0.03	0.09	0.03	0.02	0.08	0.02
Leader narcissism				0.27	0.07	0.24^***^	0.31	0.08	0.27^***^	0.28	0.08	0.25^***^
Hypercompetitive attitude X coworker IM							−0.00	0.11	−0.00^**^	0.09	0.11	0.07
Hypercompetitive attitude X leader narcissism							−0.09	0.10	−0.07	−0.05	0.10	−0.04
Coworker IM X leader narcissism							0.25	0.07	0.20^***^	0.32	0.08	0.26^***^
Hypercompetitive attitude X coworker IM X leader narcissism										0.19	0.06	0.23^***^
*F*	0.73			61.39^***^			4.15^**^			11.01^***^		
*R* ^2^	0.02			0.41			0.44			0.46^*^		
*R* ^2^				0.39			0.03			0.02^*^		

*N = 286*.

* *p < 0.05*,

** *p < 0.01*,

*** *p < 0.001*.

**Figure 2:**
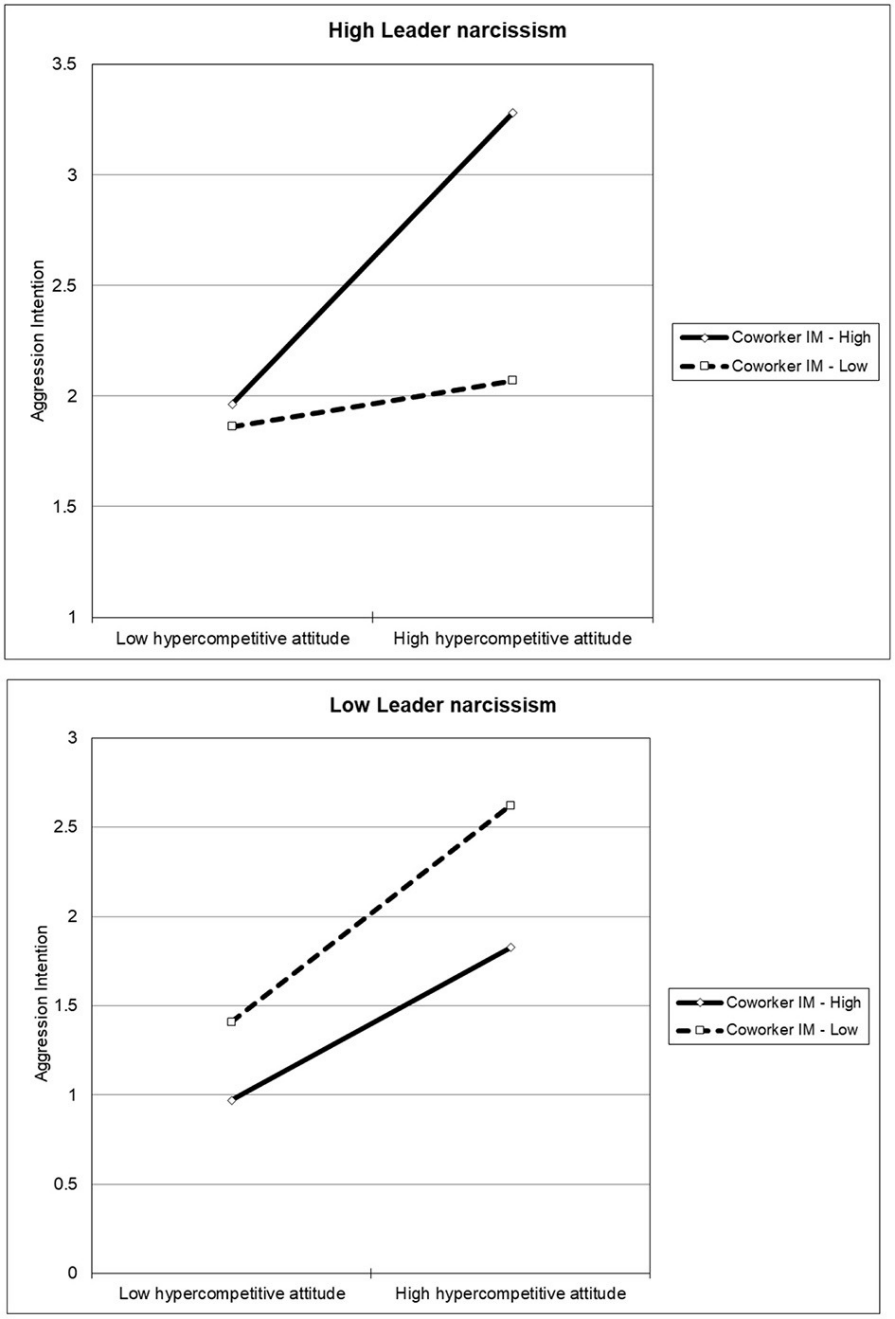
The three-way interaction of hypercompetitive attitude, coworker IM, and leader narcissism on aggression intention.

To identify the pattern of this three-way interaction effect, simple slope and slope difference tests were conducted following Aiken et al. (1991) and Dawson and Richter (2006). The results indicated that for those who perceive high levels of leader narcissism, their coworkers' IM moderated the relationship between their hypercompetitive attitude and aggression intention, in that this association was stronger among employees who perceived higher coworker IM (*B* = 0.52, *p* < 0.01) than lower (*B* = 0.30, *p* < 0.001). On the other hand, for those employees who perceived low leader narcissism, the relationship between hypercompetitive attitude and aggression intention became less strong when they recognized a higher level of coworker IM (*B* = 0.48, *p* < 0.001) than lower (*B* = 0.49, *p* < 0.001). Whether these slopes differed from each other was further examined, and the results showed that the slope differed significantly for high leader narcissism when confronted with high coworker IM as opposed to low coworker IM (*t* = 2.14, *p* < 0.05); however, the slope did not differ significantly for low leader narcissism with high coworker IM vs. with low coworker IM (*t* = −0.84, *p* = 0.40). These results indicated that the level of coworker IM is unimportant when leader narcissism is low, unlike in the case of the high leader narcissism condition. Thus, H2 was supported (Figure 2).

## Discussion

The present study contributes to the literature by highlighting the psychological mechanism in the relationship between job insecurity and aggression intention and the three-way moderating effect of hypercompetitive attitude, coworker IM, and leader narcissism. Specifically, the findings showed that job insecurity can give rise to aggression intention via hypercompetitive attitude. Additionally, the interaction effect of hypercompetitive attitude and coworker IM on aggression intention was verified only when leader narcissism is high. This study provides important theoretical and practical contributions to the workplace harassment literature by confirming and extending prior findings.

### Theoretical Implications

This study has several theoretical implications. First, this study demonstrates that employees who experience job insecurity may engage in workplace harassment intention through hypercompetitive attitude, which implies that a hypercompetitive attitude can be a signal that a focal employee may experience job insecurity and be likely to behave aggressively. The results supported that hypercompetitive attitude plays an important role in job insecurity as a mechanism for inducing workplace harassment behavior. Such investigation has rarely been confirmed by previous studies (Schilpzand et al., 2016; Lee et al., 2018). My findings suggest that increased competition awareness among employees who perceive that they may lose their jobs not only maximizes hypercompetitive attitude but also has a significant effect on workplace harassment behavior. These results provide evidence about the relationship between psychological anxiety about losing a job and harassment intention in the workplace.

Second, this study extends the workplace harassment literature by applying a novel theory to reveal the complicated relationship among the factors that produce workplace harassment. COR has been applied mostly in organization stress research. Although some research has applied COR to other organizational variables, such as absenteeism (Van Woerkom et al., 2016), turnover (Reina et al., 2018) and job performance (Park et al., 2014), few studies have used COR to examine the occurrence of workplace harassment behavior. In the existing workplace harassment research, theories such as the “cognitive activation theory of stress,” “job demands-resources theory,” and “frustration-aggression hypothesis” have been used (Rai and Agarwal, 2018). One study used COR in the workplace harassment literature (Tuckey and Neall, 2014); however, it intended to verify the effects of workplace bullying on employees' self-efficacy, rather than defining the antecedents of workplace harassment behavior. Instead, the present study confirmed the factors that affect workplace harassment based on COR theory, implying that workplace harassment can be developed through how employees manage resources. This also satisfies the call of previous research that suggested the use of COR in studies describing the relationship between antecedents and workplace harassment (Rai and Agarwal, 2018). Consequently, this study deepened the understanding of the way workplace harassment takes place.

Third, this study found that the higher an employee's hypercompetitive attitude, the more likely the occurrence of workplace harassment intention. More importantly, this relationship is manifested when both leader narcissism and coworker IM are high. In other words, high leader narcissism and high coworker IM are necessary for the transition of hypercompetitive attitude to harassment intention, indicating that high hypercompetitive attitude and high coworker IM jointly affect an individual's harassment intention, and these effects are especially valid when leader narcissism is high. Conversely, in the case of low leader narcissism, the level of coworker IM was not truly crucial to the relationship between hypercompetitive attitude and harassment intention. Given that workplace harassment behavior is closely related to leadership (Hershcovis and Barling, 2010), this research adds another characteristic of leadership, i.e., leader narcissism, that can trigger individual employees' aggression intention.

Another important finding of this study is that individual interpretations of job insecurity play an important role in the occurrence of workplace harassment intention. According to the results, anxiety about losing a job can cause workplace harassment, which signifies the need for more in-depth studies on the insecure psychological state of workers, especially in today's scenario where AI is expected to continue to replace human jobs.

### Practical Implications

This study has several important practical implications that can be considered by human resource managers, etc. First, findings of this study raise questions regarding the assumption that a competitive attitude is a significant factor in producing organization. Many companies have managed human resources in a way that improves performance by applying competitive compensation systems, inducing an unstable state among employees. However, even though such a system can improve performance, competitive attitudes can result in employee harassment intention; thus, human resource policies should be designed with the consideration that policies pertinent to reinforcing competitive attitude can have a negative effect.

Second, this study provides insights into reducing the incidence of aggression intention while still maintaining a competitive compensation system by discovering factors that worsen the negative effects of competition in the occurrence of employees' harassment intention. In a competitive situation, the more often employees perceive leader narcissism and coworker IM, the stronger aggression intention occurs. If a competitive compensation system is to be used, leaders should be educated to refrain from narcissistic behavior or to set up systems that limit narcissistic tendencies. Additionally, clearer rules of conduct within each role are needed so that employees do not consider IM as an in-role action and are more committed to actual work-related behavior.

Finally, pathways to alleviate stress in the workplace should be established. Job insecurity has already become widespread worldwide, and employees may now be more competitive than before. If an organization provides employees who experience job insecurity with a sense of perceived organizational support, or if there is a conversation window to address this anxiety, the formation of aggression intention due to job insecurity may be mitigated.

### Limitation and Directions for Future Research

Despite these implications, some limitations exist in this study. First, data was collected from a single source at a single time, which can raise concern for common method bias. However, there are several reasons why I believe that this method is suitable for this research. This study focuses on the effect of employees' subjective state on their intention. As such, it is a person's perception of their environment that is of chief importance. Measuring job insecurity, hypercompetitive attitude, and aggression intention using a non-self-report method would be inaccurate when rated from others because the most effective measurement strategy should rely on other people's inferences. Specifically, this study is based on the COR (i.e., resource use from the individuals' point of view) and the theory of psychological contract breach focusing on personal judgement, in which capturing self-appraisal about the situation is crucial. Moreover, measuring leader narcissism and IM from employees' perception is the appropriate way since these variables may not be accurately assessed given that the person directly involved may underreport their own undermining behavior (Pauls and Crost, 2004; Lee and Carpenter, 2018). It is also worth noting that interaction effects are unlikely to be influenced by a single source method (Siemsen et al., 2010). Furthermore, this study minimized common method bias by following Podsakoff et al.' 2012 suggestions. As mentioned in the Method section, participants were assured their anonymity and confidentiality of their responses and ensured that no right or wrong answers exist in order to avoid socially-desirable, lenient, or acquiescent responses. Not only items with similar contents, but also predictor and criteria questionnaires were also separately assigned. Statistical efforts including AVE and one-factor test to minimize common method bias were conducted as well. Although I have confidence in the validity of this study, future research collecting data from multiple sources and times could provide more direct evidence on the proposed model.

Second, a slight bias in sample distribution was a further limitation of this study: Given that the proportion of office workers was 74.8% and that of permanent workers was 92%, the results of this study cannot be generalized to other sectoral and temporary worker samples. Therefore, future studies should consider samples from more varied industrial sectors and employment types to increase the possibility of generalization.

Future research should also examine more diverse ways in which aggression intention occurs in the workplace. Several theories have been proposed to explain the mechanism by which workplace harassment occurs. Hutchinson et al. (2006) found that the path of bullying behavior depends on the location of power in the workplace. Until now managers have been the most influential bullies, so managers can be victims of aggression, given unofficial power. Such proposals will enrich the research on aggressive behavior in the workplace.

Finally, it is necessary to discover the further moderating factors that can soothe the process of aggression intention due to hypercompetitive attitude caused by job insecurity. For example, those with strong moral identities are more likely to take ethical actions since they have a high level of ethical cognitive competency (Aquino and Reed, 2002). Hence, the higher the level of moral identity, the less likely an individual will have aggression intentions, even if they have formed a hypercompetitive attitude due to job insecurity. Future research might provide meaningful implications regarding this issue.

## Data Availability Statement

The original contributions presented in the study are included in the article/supplementary material, further inquiries can be directed to the corresponding author.

## Ethics Statement

Ethical review and approval was not required for the study on human participants in accordance with the local legislation and institutional requirements. The patients/participants provided their written informed consent to participate in this study.

## Author Contributions

The author confirms being the sole contributor of this work and has approved it for publication.

## Funding

This study was supported by Electronics and Telecommunications Research Institute (ETRI) grant funded by the Korean Government (grant number 21ZR1420).

## Conflict of Interest

The author declares that the research was conducted in the absence of any commercial or financial relationships that could be construed as a potential conflict of interest.

## Publisher's Note

All claims expressed in this article are solely those of the authors and do not necessarily represent those of their affiliated organizations, or those of the publisher, the editors and the reviewers. Any product that may be evaluated in this article, or claim that may be made by its manufacturer, is not guaranteed or endorsed by the publisher.
